# Carbapenem-resistance and pathogenicity of bovine *Acinetobacter indicus*-like isolates

**DOI:** 10.1371/journal.pone.0171986

**Published:** 2017-02-16

**Authors:** Peter Klotz, Stephan Göttig, Ursula Leidner, Torsten Semmler, Sandra Scheufen, Christa Ewers

**Affiliations:** 1 Institute of Hygiene and Infectious Diseases of Animals, Justus-Liebig-University Giessen, Giessen, Germany; 2 Institute for Medical Microbiology and Infection Control, Hospital of the Johann Wolfgang von Goethe-University, Frankfurt am Main, Germany; 3 Robert Koch Institute, Berlin, Germany; Tianjin University, CHINA

## Abstract

The objective of this study was to characterize *bla*_OXA-23_ harbouring *Acinetobacter indicus*-like strains from cattle including genomic and phylogenetic analyses, antimicrobial susceptibility testing and evaluation of pathogenicity *in vitro* and *in vivo*. Nasal and rectal swabs (n = 45) from cattle in Germany were screened for carbapenem-non-susceptible *Acinetobacter* spp. Thereby, two carbapenem resistant *Acinetobacter* spp. from the nasal cavities of two calves could be isolated. MALDI-TOF mass spectrometry and 16S rDNA sequencing identified these isolates as *A*. *indicus*-like. A phylogenetic tree based on partial *rpoB* sequences indicated closest relation of the two bovine isolates to the *A*. *indicus* type strain A648^T^ and human clinical *A*. *indicus* isolates, while whole genome comparison revealed considerable intraspecies diversity. High mimimum inhibitory concentrations were observed for carbapenems and other antibiotics including fluoroquinolones and gentamicin. Whole genome sequencing and PCR mapping revealed that both isolates harboured *bla*_OXA-23_ localized on the chromosome and surrounded by interrupted *Tn*2008 transposon structures. Since the pathogenic potential of *A*. *indicus* is unknown, pathogenicity was assessed employing the *Galleria* (*G*.) *mellonella* infection model and an *in vitro* cytotoxicity assay using A549 human lung epithelial cells. Pathogenicity *in vivo* (*G*. *mellonella* killing assay) and *in vitro* (cytotoxicity assay) of the two *A*. *indicus*-like isolates was lower compared to *A*. *baumannii* ATCC 17978 and similar to *A*. *lwoffii* ATCC 15309. The reduced pathogenicity of *A*. *indicus* compared to *A*. *baumannii* correlated with the absence of important virulence genes encoding like phospholipase C1+C2, acinetobactin outer membrane protein BauA, RND-type efflux system proteins AdeRS and AdeAB or the trimeric autotransporter adhesin Ata. The emergence of carbapenem-resistant *A*. *indicus*-like strains from cattle carrying *bla*_OXA-23_ on transposable elements and revealing genetic relatedness to isolates from human clinical sources requires further investigations regarding the pathogenic potential, genomic characteristics, zoonotic risk and putative additional sources of this new *Acinetobacter* species.

## Introduction

*Acinetobacter baumannii* is an opportunistic pathogen frequently involved in a wide range of nosocomial infections [1]. For more than a decade now, carbapenem-resistant *Acinetobacter* spp. strains, particularly *A*. *baumannii*, represent a growing public health concern, since they often confer resistance to other critically important antimicrobials, including aminoglycosides, fluoroquinolones or even polymyxins [2, 3]. Carbapenem resistance in *Acinetobacter* spp. is most often mediated by oxacillinases (OXA) which belong to the group of carbapenem-hydrolysing class D β-lactamases (CHDLs). The most prevalent OXA carbapenemases found in *Acinetobacter* are acquired OXA-23 and OXA-58 which can be either plasmid or chromosome encoded. OXA carbapenemases exhibit only weak hydrolysis of carbapenems *in vitro* but are often associated with insertion sequences that provide additional promoter elements leading to overexpression of CHDLs and finally to carbapenem resistance in clinical isolates [1–3]. Although reports about carbapenem resistant *Acinetobacter* spp. strains in animals are still infrequent, they have been increasing in the last few years. OXA-23 was identified in *A*. *variabilis* from cattle in France [4], in *A*. *gandensis* strains from horses in the Netherlands [5], and in *A*. *baumannii* from cats in Portugal and Germany [6–8]. Different carbapenemases were further recovered in *A*. *baumannii* from livestock animals in Lebanon [9], and from swine in China [10]. In 2012, a novel species, termed *A*. *indicus*, has been identified from a cyclohexane-containing dumpsite [11]. Two years later Bonnin *et al*. reported OXA-23 mediated carbapenem resistance in a human clinical isolate identified to be closely related to this species and, thus, was termed *A*. *indicus*-like [12].

In the present study, two carbapenem-resistant, OXA-23 carrying *Acinetobacter indicus*-like isolates were recovered from nasal swabs of two calves. We characterized the genetic environment of *bla*_OXA-23_, and performed genomic and phylogenetic analyses in order to get insights into acquisition and dissemination. Since the clinical relevance of *A*. *indicus* is unknown, we evaluated pathogenicity *in vitro* and *in vivo* in comparison to reference strains of *A*. *baumannii* and the closely related *A*. *lwoffii*.

## Materials and methods

### Bacterial strains, species identification and assignment to international clones

From September 2014 to March 2015 nasal and rectal swabs as well as composite fecal samples from the corresponding stables (n = 45) were taken from cattle (*Bos taurus*) in Hesse (coordinates 50°39′58″N 8°35′28″E), Germany. Cattle breeds included Holstein-Frisian, Angus, Hereford, Swiss-Brown, Pinzgauer and Vogelsberger Rotes Höhenvieh. The samples were cultured on blood agar (blood agar base by Merck Chemicals, Darmstadt, supplemented with 5% sheep blood) and on Gassner agar (Oxoid, Wesel, Germany). Screening for carbapenem-non-susceptible *Acinetobacter* spp. was done by using Mueller-Hinton agar plates (Oxoid, Wesel, Germany) containing 2 mg/L and 4 mg/L meropenem (Sigma-Aldrich, Munich, Germany), respectively. Colonies with suspected reduced susceptibility to carbapenems were initially identified at the species level using matrix-assisted laser desorption/ionization time-of-flight mass spectrometry (MALDI-TOF MS; Bruker Daltonics, Bremen, Germany). Species identification was verified by multiplex PCR targeting different portions of the *gyrB* gene and by 16S rRNA gene sequence analysis [13].

### Whole genome sequencing and phylogenetic analysis and screening for virulence-related genes

For whole genome sequencing of two bovine *A*. *indicus* strains, DNA was extracted with the “Master Pure^™^ DNA Purification Kit” (Biozym Scientific GmbH, Hessisch Oldendorf, Germany). Genome sequencing was done using an Illumina MiSeq sequencer with multiplexing of 30 samples per flow cell using 300 bp paired-end reads and a minimum of 50-fold coverage. Sequence data were assembled de novo using SPAdes Genome Assembler V. 3.8 [14]. Phylogenetic analysis was initially performed by using the partial *rpoB* sequence of *A*. *indicus*-like strains and comparing them with publicly available *rpoB* sequences of type or reference strains of known species of the genus *Acinetobacter*. Similarity calculations and cluster analysis were carried out for a 823 bp region spanning nucleotide positions 2944–3766 of the *rpoB* coding region of *A*. *baumannii* CIP70.34^T^. MAFFT (Multiple Alignment using Fast Fourier Transform) alignment was performed to cluster partial *rpoB* sequences [15]. A PhyML (Phylogenetic software based on the Maximum-Likelihood principle) tree was created using the HKY85 substitution model and bootstrap values were determined after 1000 simulations using the Geneious 8.1.3 software (Biomatter Ltd., Auckland, New Zealand) [16].

Phylogenetic relationships were further determined on the basis of the Maximum Common Genome (MCG) [17], which represents the set of orthologous genes that are present in all genomes under study. In a first comparison, we included 32 publicly available representatives of the different *Acinetobacter* spp. (S1 Fig). Secondly, we compared the genomes of six *A*. *indicus* strains (including three genome sequences of strain A648^T^, which were submitted under different labels), namely IHIT27599 (accession number MRUS00000000), IHIT27630 (MRUT00000000), KM7 (JZRF01000070), CIP 110367 (= A648 ^T^; ACET00000000.1), ANC 4215 (= A648^T^; ATGH00000000.1), and DSM 25388 (= A648^T^; BBSF00000000.1). A prediction of genes was performed by using the Prokaryotic Dynamic Programming Genefinding Algorithm For Microbial Genomes (Prodigal) [18]. The coding sequences where subsequently clustered using USEARCH v7 [19] based on a threshold of 70% similarity on nucleotide level and 90% coverage to determine the set of orthologous genes of all genomes included in the respective comparison. Using these sets as a reference, we extracted the corresponding allelic variants of the MCG genes from the genomes (32 and 6, respectively) using PLAST v2.3.1 [20], aligned them with MUSCLE v3.8.31 [21] and finally concatenated them. The resulting alignment was used to infer a maximum likelihood phylogeny using RAxML version 8.1.14 with a General Time Reversible model and gamma correction for among site rate variation [22].

### Antimicrobial susceptibility and resistance genes

Antimicrobial susceptibility was determined by antibiotic gradient tests (Liofilchem, Roseto degli Abruzzi, Italy). Minimum inhibitory concentrations (MICs) were interpreted according to breakpoints defined for human *Acinetobacter* spp. by either EUCAST or CLSI [23, 24]. MICs of tigecycline and chloramphenicol were interpreted according to breakpoints for Enterobacteriaceae set by EUCAST [23]. Whole genome sequences were used to identify resistance genes by using the online tool ResFinder 2.1, provided by the Center for Genomic Epidemiology (CGE) (http://www.genomicepidemiology.org/). PCR mapping of the genetic environment of *bla*_OXA-23_ was performed to determine the correct order of contigs and sequence assemblies using primers listed in S1 Table. The genetic location of the *bla*_OXA-23_ gene was evaluated by performing I-*Ceu*I digestion of whole-cell DNAs followed by Southern blot hybridization using 16S rRNA and *bla*_OXA-23_ probes as previously reported [25, 26].

### Analysis of pathogenicity in the *Galleria mellonella* infection model

Pathogenicity of *Acinetobacter* spp. strains was analysed employing last-instar larvae of the greater wax moth (*Galleria mellonella*) [27]. A 1:50 dilution of an overnight bacterial culture in lysogeny broth (LB) was prepared and grown to an OD_600_ of 1.0 at 37°C. A phosphate-buffered saline (PBS) solution containing serial dilutions of this culture representing colony forming units of 5x10^2^ to 5x10^6^ was injected into the last left proleg of the larvae using a Hamilton precision syringe. PBS solution alone served as negative control. Upon infection, larvae were incubated in petri dishes at 37°C for 72 h and scored for survival by two independent observers daily. For determination of the median lethal dose (LD_50_), a series of 10-fold serial dilutions were injected and LD_50_ were calculated after 24 h by nonlinear regression analysis using GraphPad Prism 5.0 (La Jolla, USA) as described [28, 29].

### Cell viability assay

A549 human lung epithelial cells (ATCC^®^ CCL-185) were grown in six well plates in Dulbecco’s Modified Eagle Medium (DMEM; Biochrom GmbH, Berlin, Germany) with 10% foetal calf serum (FCS; Biochrom GmbH, Berlin, Germany) at 37°C until almost confluent. Different *Acinetobacter* spp. were used at a multiplicity of infection (MOI) of 100 and incubated for 20 h. Thereafter, the supernatant was filtered (0.45 μm) and lactate dehydrogenase (LDH) activities were determined by spectrophotometry at a wavelength of 340 nm using the IFCC method as described by Schuman *et al*. [30]. The detergent Triton X-100 (0.1% in PBS) and DMEM were used as positive and negative controls, respectively. Mean LDH values of medium-treated A549 cells versus infected cells were analysed by an unpaired two-tailed Student’s *t* test (Graph Pad Prism 5.0). A *p* value of ≤0.05 was considered statistically significant, and a *p* value of ≤0.001 was considered highly significant.

### Screening for virulence-related genes

Screening for virulence-related genes was performed by using the online tool MyDbFinder 1.1, provided by the Center for Genomic Epidemiology (https://cge.cbs.dtu.dk/services/). Only genes that have previously been associated with one of the phenotypes investigated later, i.e. killing of *G*. *mellonella* larvae and cytotoxicity to human lung epithelial cells, were included. Using the Geneious 8.1.3 software a MAFFT alignment was performed to cluster nucleotide/amino acid sequences and to calculate sequence identity to a given reference sequence.

## Results

### Susceptibility of bovine *Acinetobacter* spp. isolates to carbapenems

During screening of cattle for carbapenem-non-susceptible *Acinetobacter* spp. two isolates showed growth on the meropenem-containing screening agar. Strain IHIT27630 was isolated in September 2014 from the nasal cavity of a calf which was hospitalized in a veterinary clinic due to dermatophytosis, wasting syndrome, diarrhoea and bronchopneumonia. The second strain, IHIT27599, was isolated one month later from the nasal cavity of a calf which was suffering from bronchopneumonia and omphalophlebitis. This calf was sampled on a farm 35 miles away from the veterinary clinic and nearly 60 miles apart from the farm where the first calf originated from, making an epidemiologic link and a transmission highly unlikely. Using MALDI-TOF MS analysis, both isolates were presumably suggested as *A*. *calcoaceticus* but with low reliability (score values of 1.57 and 1.56 using the IVD MALDI Biotyper library). 16S rDNA sequence analysis enabled a more precise identification and indicated a high homology (>99%) to published sequences of *A*. *indicus* and *A*. *indicus*-like strains, including strain RAB1 and the type strain A648^T^. RAB1 originates from a human rectal swab and was isolated in France in 2011, whereas A648^T^ was obtained from a cyclohexane dumping site in India sometime before 2010 [12, 31].

### Phylogenetic analysis and genome comparison

**Fig 1:**
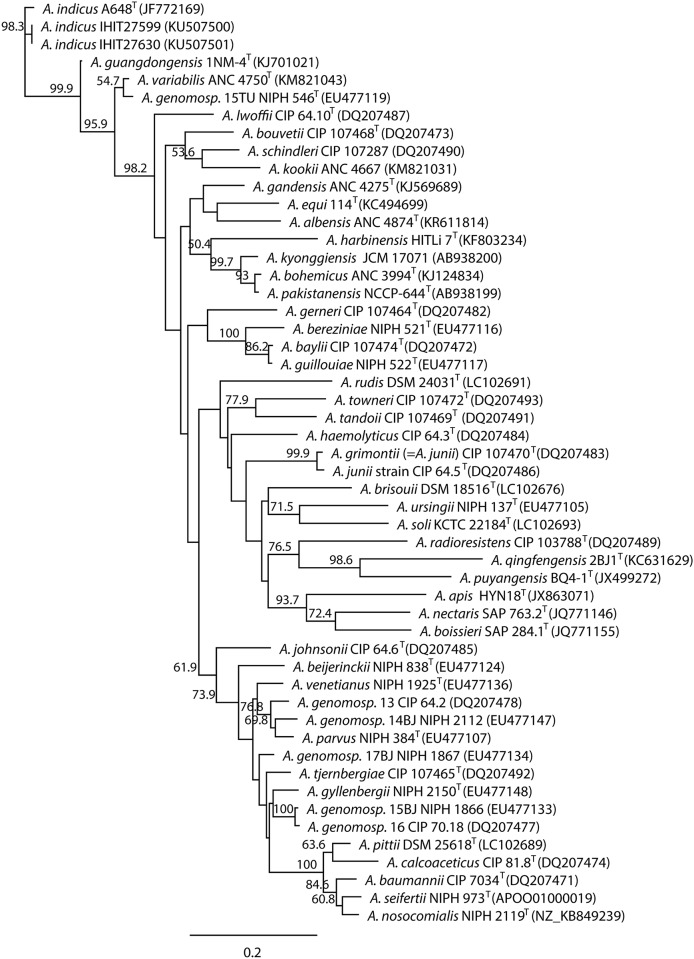
Neighbour-joining phylogenetic tree based on partial nucleotide sequences of the *rpoB* (823 bp) genes of *Acinetobacter indicus*-like strains IHIT27630 and IHIT27599 and 47 type or reference strains of known *Acinetobacter* species. The tree was constructed using the maximum likelihood method. Bootstrap values (> 50%) after 1,000 simulations are shown at branch nodes. GenBank accession nos. are given in parentheses. Bar, 0.2 nucleotide substitutions per site.

**Fig 2:**
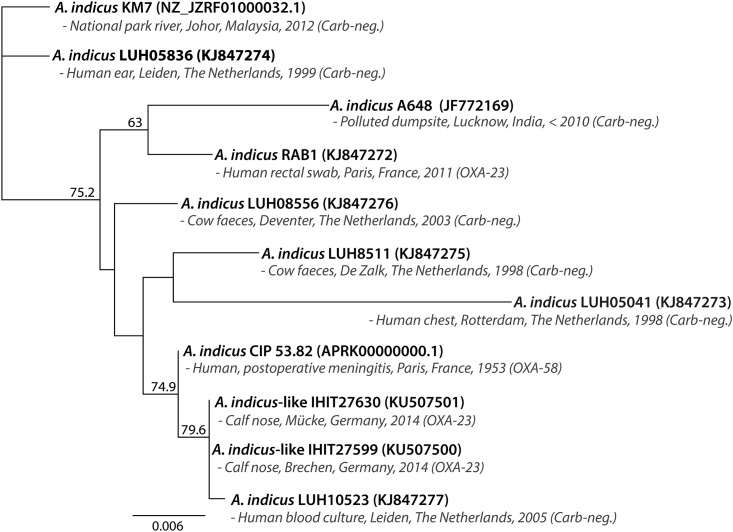
Neighbour-joining phylogenetic tree based on partial nucleotide sequences of the *rpoB* (823 bp) genes of *Acinetobacter indicus*-like strains IHIT27630 and IHIT27599 and nine published *A*. *indicus* strains, including relevant information about original host, geographical background, year of isolation and possession of carbapenemases. The tree was constructed using the maximum likelihood method. Bootstrap values (> 50%) after 1,000 simulations are shown at branch nodes. GenBank accession nos. are given in parentheses. Bar, 0.006 nucleotide substitutions per site. Carb-neg., carbapenemase negative.

A phylogenetic tree was compiled based on the alignment of partial 823-bp *rpoB* sequences from the two bovine *A*. *indicus-like* isolates, 34 distinct *Acinetobacter* spp. with validly published names, seven *Acinetobacter* spp. with effectively published names awaiting validation (www.bacterio.net), and six *A*. *genomospecies* strains. The analysis indicated closest relation between IHIT27630 and IHIT27599 and the *A*. *indicus* type strain A648^T^ with 97.69% nucleotide sequence similarity of partial *rpoB* sequence (Fig 1). The next closest related strains were *A*. *guangdongensis* strain 1 NM-4^T^ (92.22%), *A*. *variabilis* ANC 4750 (87.97%), *A*. *genomosp*. 15TU (now *A*. *variabilis*) NIPH 546 (86.76%), and *A*. *lwoffii* CIP 61.10^T^ (86.39%), while *A*. *qingfengsis* strain 2BJ1^T^ revealed the least closely related *rpoB* sequence (76.01%). When generating a phylogenetic tree employing all published *rpoB* sequences from *A*. *indicus*-like strains (accessed at 29^th^ December 2016), our bovine isolates clustered with human clinical *A*. *indicus*-like strain LUH10523 (99.88% nucleotide sequence identity) that was obtained from the blood culture of a patient in The Netherlands in 2005 (Fig 2) [12]. Next closely related was *A*. *indicus*-like strain CIP 53.82 that was obtained from a human patient with postoperative meningitis in 1953 in France [32, 33]. Overall, the *rpoB* regions showed intraspecies similarity values for the 11 strains, ranging between 96.36% and 100%. There was no evidence for a separation of environmental (strains KM7 and A648^T^), animal (isolates from the present study and strains LUH08556 + LUH8511 from cow faeces) and human strains (LUH05836, RAB1, LUH05041 and LUH10523) or of strains with or without carbapenemases [12]. Apart from our study isolates, only *A*. *indicus*-like strain RAB1 expressed the OXA-23 carbapenemase [12]. In addition, *in silico* analysis of the genome sequence of *A*. *indicus*-like strain CIP 53.82 (acc. no. APRK00000000.1) revealed the presence of the *bla*_OXA-58_ gene, which is flanked by two incomplete IS*Aba3* insertion elements [32, 33].

For a higher phylogenetic resolution, we further compared 32 isolates of different *Acinetobacter* spp. based on genome sequences (S1A Fig). As whole genomes of several species included in the *rpoB*-based tree (Fig 1) were not available, a direct comparison of both tree phylogenies was not possible. The MCG of the 32 selected *Acinetobacter* spp. isolates revealed 42 orthologous genes and the alignment of the genes consisted of 26,161 sites from which 8,026 were informative SNP sites. Pairwise distance varied between 0 and 4,049 sites. Similar to what has been observed for the *rpoB*-based tree, the *A*. *indicus* strains clustered together and strains of the species *A*. *towneri*, *A*. *tandoii* were placed in closer vicinity as for example those of *A*. *baumannii* and *A*. *pittii*, as shown in S1A Fig and verified by pairwise distance values (S2 Table).

A separate calculation of the MCG for the *A*. *indicus* strains revealed 2,145 orthologous genes and the alignment of these genes consisted of 2,027,793 sites from which 109,137 were phylogenetically informative SNP sites. Based on this analysis, the genome sequences submitted under three different labels and accession numbers for *A*. *indicus* type strain A648^T^ differed by 313 (ANC 4215 versus CIP 110367) to 1917 (CIP 110367 versus DSM 25388) SNPs, which may be due to different sequencing strategies or strain material. Apart from this, hardly any similarity-based clustering could be observed, suggesting a considerable diversity between the genomes of strains A648^T^, KM7, IHIT27599 and IHIT27630. This was also evident in the pairwise distance which varied between 64,841 and 86,573 SNPs to the type strain (S2B Table). Even the two bovine IHIT strains differed by 35,396 from each other, clearly indicating two different strains.

### Antimicrobial susceptibility and resistance genes

The two bovine *A*. *indicus*-like isolates showed high MICs to imipenem, meropenem and doripenem (Table 1) and represent the first known carbapenem-resistant *A*. *indicus*-like isolates of animal origin. While the isolates remained susceptible to third and fourth generation cephalosporins, non-susceptibility was observed for a number of other antimicrobial substances, including piperacillin-tazobactam, fluoroquinolones, gentamicin, and doxycycline (Table 1). The two isolates differed slightly in their antimicrobial resistance profile with IHIT27599 being additionally resistant to tobramycin and co-trimoxazole. Sanger sequencing confirmed that both isolates harboured the β-lactamase OXA-23 which is widespread in *A*. *baumannii* [34]. Subsequent whole genome sequencing revealed the presence of aminoglycoside resistance genes *aac(3)-IIa*, *strA/B* and *aph(3’)-Ic*, sulfonamide resistance gene *sul2*, phenicol resistance gene *floR*, and tetracycline resistance genes *tet*(A) and *tet*(Y) in isolate IHIT27630. The second isolate (IHIT27599) possessed aminoglycoside resistance genes *aadA1*, *aadB*, *strA*/*B* and *aph(3`)-Ic*, sulfonamide resistance genes *sul1* and *sul2*, phenicol resistance gene *floR*, and tetracycline resistance genes *tet*(X) and *tet*(Y), which is in line with the phenotypic results.

**Table 1:** Antimicrobial susceptibility of bovine *Acinetobacter indicus*-like isolates.

		IHIT27630		IHIT27599	
Drug class	Antimicrobial agent	MIC (mg/L)	Suscep-tibility^a^	MIC (mg/L)	Suscep-tibility^a^
Penicillins + inhibitors	Piperacillin	>256	R	64	I
	Ampicillin/sulbactam^b^	2	S	8	S
	Piperacillin/tazobactam^b^	>256	R^H^	64	I
	Ticarcillin/clavulanic acid	>256	R^H^	>256	R
Cephalosporins	Cefotaxime	4	S	2	S
	Ceftriaxone	4	S	2	S
	Ceftazidime	2	S	4	S
	Ceftiofur	4	I	4	I
	Cefepime	4	S	2	S
Carbapenems	Imipenem	>32	R^H^	32	R
	Meropenem	>32	R^H^	32	R
	Doripenem	8	R^H^	16	R
Fluoroquinolones	Ciprofloxacin	>32	R^H^	4	R
	Levofloxacin	>32	R^H^	4	R
Aminoglycosides	Gentamicin	16	R	8	R
	Amikacin	0.5	S	1	S
	Tobramycin	2	S	8	R
	Netilmicin	4	S	1	S
Tetracyclines	Tetracycline	32	R	128	R
	Doxycycline	16	R	16	R
	Minocycline	1	S	0.5	S
Chloramphenicol	Chloramphenicol	64	R	128	R
Glycylcycline	Tigecycline	0.5	S	0.125	S
Folate pathway inhibitors	Trimethoprim/sulfamethoxazole	1	S	>32	R
Polymyxins	Polymyxin B	1	S	1	S
	Colistin	1	S	1	S

S, susceptible; I, intermediate susceptible; R, resistant; H, heteroresistance.

^a^MICs were interpreted according to breakpoints for *Acinetobacter* spp. set by EUCAST and CLSI. MICs for tigecycline and chloramphenicol were interpreted according to breakpoints for Enterobacteriaceae set by EUCAST. MICs for Ceftiofur were interpreted according to breakpoints of 3^rd^-generation cephalosporines set by the CLSI for *Acinetobacter* spp.

^b^Beta-lactamase inhibitors sulbactam and tazobactam were used with a fixed concentration of 4 mg/L as recommended by EUCAST.

### Localization and genetic environment of *bla*_OXA-23_

**Fig 3:**
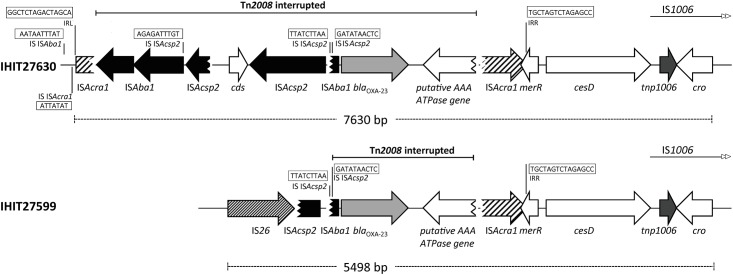
Schematic maps of the genetic environment of *bla*_OXA-23_ in bovine *A*. *indicus*-like strains. The interrupted Tn*2008* is indicated with a solid line. Target site duplications are likely missing due to other insertion events, such as IS*Acsp2* at the left border and IS*Acra1* at the right border of Tn*2008*. IS, insertion site; IRL, inverted repeat left; IRR, inverted repeat right; *ATPase* (truncated), gene encoding the putative AAA ATPase; *merR*, gene encoding the transcriptional regulator MerR; *cesD*, gene encoding the cobalt-zinc-cadmium resistance protein CesD; *cro*, gene encoding the Cro-like protein; *cds*, encoding a hypothetical protein. The figure is not to scale. GenBank accession nos. KU833218 (IHIT27599) and KU833219 (IHIT27630).

I-*Ceu*I digestion of whole-cell DNAs and Southern blot hybridization using 16S rRNA and *bla*_OXA-23_ probes demonstrated that both *A*. *indicus*-like isolates harboured *bla*_OXA-23_ on the chromosome. Genome sequencing and PCR mapping of assembled contigs identified a differently interrupted and incomplete *Tn*2008 transposon structure surrounding the OXA genes (Fig 3). In both isolates the same genetic structure is present downstream of *bla*_OXA-23_, including a putative AAA *ATPase* gene disrupted by a partial insertion sequence IS*Acra1* of 564 bp in length and the transcriptional regulator gene *merR*. We identified a 105-bp long IS*Aba1*-remnant which was either truncated by a full version of insertion sequence IS*Acsp2* in isolate IHIT27630 or by a partial IS*Acsp2* sequence which was preceded by IS*26* in isolate IHIT27599. In case of IHIT27630, a second disrupted copy of IS*Acsp2* was preceded by a full-length copy of a Tn*2008*-related IS*Aba1* transposase gene and the remaining 168-bp sequence of the aforementioned truncated IS*Acra1*. IS*Acra1* is a novel insertion element that has previously been described in an *A*. *radioresistens* strain where it was flanked by a typical 7-bp direct repeat (DR) / insertion site (ATTATAT) as well as a 15-bp inverted repeat left (IRL; GGCTCTAGACTAGCA) and inverted repeat right (IRR; TGCTAGTCTAGAGCC) [35]. In isolate IHIT27630, the disrupted IS*Acra1* element lacked the downstream DR, whereas the characteristic upstream DR and the two IR regions were identical to those described recently [35]. In both isolates, 27 nucleotides are present between insertion sequence IS*Aba1* and the start codon of *bla*_OXA-23_, which is typical of Tn*2008* in contrast to the previously described Tn*2008b* [36].

Different genetic structures associated with the *bla*_OXA-23_ genes in the two *A*. *indicus* isolates supports previous reports about the high variability of this flanking region in *A*. *baumannii*. In addition, the finding of ISA*cra1*, although disrupted, may be a hint towards *A*. *radioresistens* as original source of the *bla*_OXA-23_ gene not only in *A*. *baumannii* but also in *A*. *indicus* [37].

### Virulence properties of *A*. *indicus*-like strains

**Fig 4:**
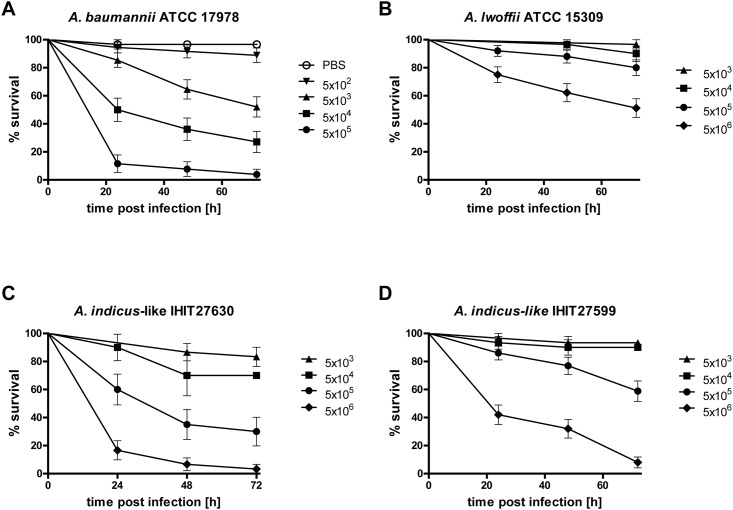
Dose-dependent lethality of *Galleria mellonella* infected with *Acinetobacter* spp. strains. Larvae were injected with different cfu (5x10^2^ to 5x10^6^) and survival was monitored over 72 h after infection. Mean values from at least four experiments are shown. Error bars show standard error of the mean.

Clinically relevant biological features of *A*. *indicus* remain totally elusive. In order to evaluate the virulence of IHIT27599 and IHIT27630, the *Galleria mellonella in vivo* infection model was employed. *G*. *mellonella* larvae were infected with different infection doses of the two bovine *A*. *indicus*-like isolates and reference strains of *A*. *baumannii* (ATCC 17978) and *A*. *lwoffii* (ATCC 15309), which were included for comparative analysis. Infection of larvae with the different *Acinetobacter* spp. caused a time- and dose-dependent killing of larvae (Fig 4). Whereas almost no mortality was observed when 5x10^4^ cfu of *A*. *indicus*-like species were injected, almost 100% of *G*. *mellonella* larvae died 72 h post infection with 5x10^6^ cfu (Fig 4C and 4D). Injection of the highest dose of 5x10^6^ *A*. *lwoffii* ATCC 15309, which was selected due to its close phylogenetic relatedness to *A*. *indicus* (Fig 1), resulted in death of approximately 40% of larvae after 72 h. In contrast, injection of only 5x10^4^ *A*. *baumannii* ATCC 17978 resulted in the death of 50% of larvae 24 h post infection. We determined median lethal doses (LD_50_) to compare virulence across the *Acinetobacter* strains (S3 Table). *A*. *indicus*-like strains IHIT27630 and IHIT27599 displayed LD_50_ values of 5.67 [95% CI 5.51–5.82] and 6.20 [95% CI 5.91–6.50] and, thus, were more virulent than *A*. *lwoffii* ATCC 15309 with an LD_50_ of 6.84 [95% CI 6.65–7.04]. *A*. *baumannii* ATCC 17978 displayed the lowest LD_50_ of 4.72 [95% CI 4.42–5.01) and was therefore the most virulent of all tested strains in the *Galleria in vivo* infection model.

**Fig 5:**
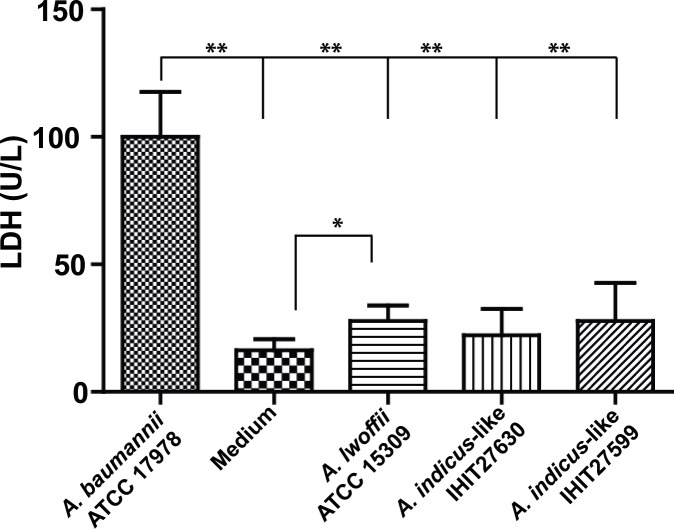
*Acinetobacter* spp. mediated cytotoxicity towards A549 human lung epithelial cells. Confluent A549 monolayers were infected for 20 h with an MOI of 100 with the indicated bacteria. Supernatants were harvested and LDH activities determined. Mean value for Triton X-100 (positive control) was 924.0 ± 93.7 U/L. Medium (DMEM) was used as a negative control. Shown are mean values ± standard deviations from n = 3–4 experiments. Asterisks indicate a significant difference between the control group (medium) and strains, respectively (* p<0.05; **p<0.001).

To investigate the capability to disrupt membrane integrity of human cells, A549 lung epithelial cells were infected with the same *Acinetobacter* strains used in the *Galleria* assay (Fig 5). Infection with *A*. *baumannii* ATCC 17978 resulted in a LDH release of 100 U/L. In contrast, infection with *A*. *lwoffii* ATCC 15309 or the two *A*. *indicus*-like strains induced a LDH release of approximately only 25 U/L (range 22.2–27.9 U/L) compared to the medium control (DMEM) (16.3 U/L) (*p* = 0.0026 for *A*. *lwoffii*; *p* = 0.0709 for IHIT27599; *p* = 0.20 for IHIT27630). This indicates that cytotoxicity towards A549 human lung epithelial cells of the three *Acinetobacter* non-*baumannii* strains was comparable to each other but much lower compared to *A*. *baumannii* ATCC 17978 (3.6 to 4.5-fold lower LDH activities; *p*<0.001).

### Presence of virulence genes related to *in vivo* virulence and *in vitro* cytotoxicity

We screened the genomes of the strains *A*. *baumannii* ATCC 17978, *A*. *lwoffii* ATCC 15309, *A*. *indicus* IHIT27599 and *A*. *indicus* IHIT27630, all of which were included in phenotypical assays, for virulence genes that have previously been linked with killing of *G*. *mellonella* larvae and cytotoxicity to human epithelial cells. *A*. *baumannii* ATCC 17978 harboured most of the virulence determinants linked with the above mentioned phenotypes and only lacked the phospholipase D1 gene *pld1* and DNA uptake protein genes *comEC* (Table 2). In contrast, *A*. *lwoffii* strain ATCC 15309 and the two *A*. *indicus* strains IHIT27599 and IHIT27630 harboured only some of the tested virulence-associated determinants but lacked for example phospholipase genes *plc1* and *plc2*, acinetobactin outer membrane receptor protein gene *bauA*, efflux pump system protein genes *adeRS* and *adeAB*, and the trimeric autotransporter adhesion gene *ata*. In case orthologous proteins could be identified in the *A*. *indicus* strains, they revealed amino acid sequence identity to the *A*. *baumannii* reference genomes ranging from 47.6% for SurA1, 54.4% for PLD1, and 62.6% for Omp33 to a maximum of 91.4% for SodB (Table 2).

**Table 2:** Presence and absence of virulence gene/protein orthologs in four *Acintebacter* species strains used for virulence assays.

Virulence determinant	Gene	Gene size in reference sequence (bp)	Gene position in reference sequence*	Nucleotide/amino acid sequence similarity to reference sequence* (%)			
				*A*. *baumannii* ATCC 17978	*A*. *lwoffii* ATCC 15309	*A*. *indicus* IHIT27599	*A*. *indicus* IHIT27630
Phospholipase C1	*plc1*	2229	1575962..1578190	100/100	**-**	**-**	**-**
Phospholipase C2	*plc2*	2169	3794856..3792688	100/100	**-**	**-**	**-**
Phospholipase D1	*pld1*	1527	140863..142389	**-**	57.1/50.3	58.6/54.4	58.6/54.4
Phospholipase D2	*pld2*	1626	524407..522783	100/100	64.2/70.4	65.9/67.1	65.9/67.1
Phospholipase D3	*pld3*	1464	628527..627064	100/100	67.4/69.4	67.3/68.4	67.2/68.2
Acinetobactin outer membrane receptor protein	*bauA*	2190	160673..158484	100/100	**-**	**-**	-
Type VI secretion system protein	*tssM*	3825	2445726..2441902	100/100	66.0/66.1	71.3/77.8	71.5/77.7
Autotransporter adhesin	*ata*	5622	2775454..2781075	100/100	**-**	**-**	**-**
Surface antigen protein 1	*surA1*	318	2349708..2350025	100/100	**-**	58.2/48.6	58.2/47.6
Efflux pump system protein	*adeRS*	1861	1929381..1931241	100/100	**-**	**-**	**-**
Efflux pump system protein	*adeAB*	4298	1931387..1935684	100/100	**-**	**-**	**-**
Efflux pump system protein	*arpA*	1101	40488..41588	100/100	66.5/70.6	66.9/71.4	67.2/71.7
Efflux pump system protein	*arpB*	3126	41591..44716	100/100	73.1/83.2	73.4/84.3	73.4/84.2
Superoxide dismutase B	*sod2343*	627	1256650..1256024	100/100	83.9/89.9	84.1/91.4	84.4/91.4
DNA uptake channel proteins	*comEC*	4198	4695..8892	**-**	**-**	**-**	**-**
Outer membrane protein A	*ompA*	1071	680965..682035	100/100	81.6/84.3	82.6/87.4	82.6/87.4
Outer membrane protein 33	*omp33*	900	212171..211272	100/100	66.1/58.8	68.8/62.6	68.8/62.6

*Refers to *A*. *baumannii* ATCC 17978 (Acc-No. CP012004.1) except for genes *pld1* (*A*. *baumannii* ATCC 19606; Acc. No. ACQB01000015.1) and *comEC* (*Acinetobacter* species BD413; AF027189.3); minus (-) denotes that the gene/protein is not present in the data source.

## Discussion

Since its first description as a novel environmental *Acinetobacter* species in 2012, very few studies reported about the isolation of *A*. *indicus*-like strains from different sources [12, 33, 38]. Difficulties in discriminating this novel species, together with its unknown clinical relevance might be reasons for that. Based on MALDI-TOF MS analysis, which has been shown to be a useful tool for identification of *Acinetobacter* spp. [32], our bovine isolates were initially misclassified as *A*. *calcoaceticus*, albeit with unreliable score values. Having similar problems with the species designation based on biochemical properties, Bonnin *et al*. (2014) established MALDI-TOF MS reference spectra for reliable identification of *A*. *indicus*-like isolates. Using these new spectra, two animal and three human *A*. *indicus*-like isolates from the Netherlands and Belgium, which were identified by *rpoB* sequencing and 16S rRNA analysis, grouped together but were clearly distinct from the type strain A648^T^ [12]. This was also evident from a genome-based comparison, where the two bovine *A*. *indicus*-like strains from the present study clustered together, but were clearly separated from the two environmental *A*. *indicus* strains A648^T^ and KM7. Future genomic studies, including a broader set of *A*. *indicus*-like isolates from different sources should help to elucidate the phylogenetic relatedness among members of this novel species more precisely, probably leading to the identification of different genotypic lineages.

Prior to our study, the first and still only published case of carbapenem resistance in an *A*. *indicus*-like strain was reported in 2014 by Bonnin *et al*. [12]. Here, the presence of OXA-23 in a rectal swab isolate (RAB1) from a French patient previously hospitalized in Algeria after a road traffic accident in August 2011 could be demonstrated [12]. In addition, we could identify the carbapenemase gene *bla*_OXA-58_ in the genome sequence of the human clinical *A*. *indicus*-like isolate CIP 53.82. Despite the presence of *bla*_OXA-58_ it is unclear, whether this strain was also phenotypically resistant to carbapenems. When evaluating antimicrobial susceptibilities for human isolate RAB1 and our bovine *A*. *indicus*-like isolates, carbapenem MICs ranged from 8 to >32 mg/L, indicating a high level resistance in both cases. Indeed, a strong promoter with distinct -10 and -35 motifs located within the IS*Aba1* sequence was present in our bovine isolates and most likely conferred clinically relevant resistance to carbapenems [39, 40]. Four other previously published *A*. *indicus*-like isolates, obtained from cow faeces and from human clinical samples between 1998 and 2003 in the Netherlands did not harbour a carbapenemase gene, nor were they resistant to carbapenems [12].

In contrast to the study of Bonnin *et al*., where *bla*_OXA-23_ of human *A*. *indicus*-like strain RAB1 was located on a conjugative plasmid, the bovine isolates from our study harboured the gene on the chromosome [12]. However, as the genomes of *A*. *indicus* strains IHIT27599 and IHIT27630 revealed plasmid sequences in the genetic surrounding of *bla*_OXA-23_, it may be assumed that the resistance gene was once acquired by horizontal plasmid transfer and subsequent loss of the plasmid, which warrants further investigations. While strain RAB1 possessed IS*Aba4* upstream of the *bla*_OXA-23_ gene, which corresponds to transposon Tn*2007* [36], the bovine isolates revealed an interrupted Tn*2008* with a single IS*Aba1* located upstream of the *bla*_OXA-23_ gene, as recently described for other isolates as well [12, 32]. Transposon Tn*2008*, which is a major vehicle for spreading of *bla*_OXA-23_ in *A*. *baumannii* [41], was recently also identified in one out of nine OXA-23 positive *A*. *variabilis* isolates from cows [4]. In several of these nine isolates, including those with the highest MICs to carbapenems, the IS*Aba1* element of Tn*2008* was truncated by a novel insertion sequence termed IS*Acsp2* [4]. In our bovine strain IHIT27630 we identified a full copy of IS*Acsp2*, preceded by an open reading frame encoding a protein of unknown function and a second truncated IS*Acsp2*. In case of IHIT27599, the truncated IS*Aba1* was directly preceded by a truncated IS*Acsp2*, indicating, that the genetic context of the *bla*_OXA-23_ gene is not unique and probably undergoes evolutionary changes.

We further detected an interrupted version of the recently described 732-bp insertion sequence element IS*Acra1* of *A*. *radioresistens* in strain IHIT27630 (Fig 3). IS*Acra1* has been associated with overexpression of the intrinsic *bla*_OXA-23_ in *A*. *baumannii* and *A*. *radioresistens* strains resulting in phenotypic carbapenem resistance [35]. This novel insertion sequence element has also been associated with the spread of *bla*_OXA-23_ in strains of the species *A*. *radioresistens*, which is the likely source of this gene [37], and this might be the case for *A*. *indicus* isolates as well.

Due to its novelty, barely anything is known about the pathogenicity of members of the species *A*. *indicus*. In order to assess pathogenicity of *A*. *indicus in vivo*, *Galleria mellonella* larvae were employed. *G*. *mellonella* has recently been described as a non-vertebrate infection model for studying human pathogens, including *A*. *baumannii* with regard to pathogenetic and therapeutic aspects [27]. In our study, both *A*. *indicus*-like strains were slightly more virulent compared to *A*. *lwoffii* ATCC 15309 since lower LD_50_ values were obtained and more larvae died when injecting same colony forming units. In contrast, *A*. *baumannii* ATCC 17978 was clearly more virulent than all other species in concordance with the LDH assay.

We further employed the LDH assay to monitor cytotoxicity towards human lung epithelial cells *in vitro*. Here, the two bovine *A*. *indicus*-like isolates and *A*. *lwoffii* displayed an abrogated phenotype in the cell toxicity assays compared to ATCC 17978 since LDH values were only slightly higher than the negative control DMEM. As anticipated, cytotoxicity of *A*. *baumannii* ATCC 17978 was approximately four times higher compared to the other *Acinetobacter* species (*p*<0.001). These results suggest that *A*. *indicus*-like strains are not cytotoxic which is mainly caused by secreted toxins or virulence factors of the outer membrane. By using knock-out mutants or regulating gene expression previous studies could identify various factors of *Acinetobacter* species to be involved in either virulence to *G*. *mellonella* larvae or cytotoxicity to epithelial cells or both. These factors include the phospolipases C (PLC) [42], phospholipases D (PLD) [43], trimeric autotransporter adhesin Ata [44], acinetobactin outer membrane receptor protein BauA [45], type VI secretion system protein TssM/VasK [46], surface antigen protein 1 (SurA1) [47], RND-type efflux system proteins AdeAB, AdeRS and ArpAB [48, 49], a superoxide dismutase (SodB) [50], and the putative DNA uptake channel protein ComEC [51]. The presence of most of the genes in the genome of *A*. *baumannii* strain ATCC 17978 corresponds well with its phenotype *in vitro* and *in vivo*, namely high cytotoxicity to epithelial cells and high ability to kill *G*. *mellonella* larvae. The absence of several factors, including PLC1 and PLC2, BauA, Ata, AdeRS, and AdeAB in *A*. *lwoffii* strain ATCC 15309 and the two *A*. *indicus* strains might—at least partially—explain the abrogated pathogenicity phenotype in both assays. Although our *A*. *indicus* strains and the *A*. *lwoffii* strain ATCC 15309 strain harboured orthologous genes/proteins of some of these factors, including PLD1-3, TSSM, ArpAB, OmpA, and Omp33, they may have lost their suggested function due to sequence alterations. Complementation of *A*. *indicus* strains with virulence factors from *A*. *baumannii* and comparative analysis of pathogenicity *in vitro* and *in vivo* with the isogenic ancestor will help to identify crucial virulence determinants in future studies.

Taken together, the two *A*. *indicus*-like strains IHIT27630 and IHIT27599 showed a comparable pathogenicity with *A*. *lwoffii* ATCC 15309 which is considered to be a rather low to moderate pathogen. Thus, *A*. *indicus* might be considered to be less pathogenic to animals and humans. However, this remains yet unknown for *A*. *indicus*-isolates from other sources and should be part of future genomic and functional studies, particularly as this novel species has already been associated with human infection. The fact that cattle are colonized with carbapenem-resistant strains harbouring *bla*_OXA-23_ on transposable elements requires further investigations regarding the zoonotic risk of this new *Acinetobacter* species. One animal which carried the OXA-23 producing *A*. *indicus*-like isolate was treated with benzyl-penicillin before, while nothing is known about antibiotic treatment of the other calf. As OXA-23 confers high-level resistance to penicillins and penicillin-β-lactamase inhibitor combinations it can be assumed, that the use of penicillins has created a selective pressure.

Even if the suggested pathogenicity of the isolates is rather low, the potential to contribute to the dissemination of the *bla*_OXA-23_ gene requires careful consideration. Studies are needed to further explore the cattle population as putative source of carbapenem resistant *Acinetobacter* spp. strains and of carbapenem resistance determinants to understand spreading of both resistance genes and carbapenem-resistant *Acinetobacter* species.

## Supporting information

S1 Table Primers and their positions used for mapping of the *bla*_OXA-23_ genetic region in bovine *A*. *indicus*-like isolates IHIT27599 and IHIT27630. (DOCX)

S2 Table SNP matrix referring to S1(A) Fig. Colour shading indicates SNP values (low number of SNPs [dark green] to high number of SNPs [white]).
(XLSX)

S3 Table Median lethal doses (LD50) of *Acinetobacter* spp. injected into *G*. *mellonella* larvae at 24 hours post infection. CI, confidence interval.
(DOCX)

S4 Table LDH statistics. (XLSX)

S1 Fig Neighbour-joining phylogenetic tree based on (A) the maximum common genome (MCG) of publicly available whole genomes of 32 representative isolates of different *Acinetobacter* species and (B) the MCG of whole genomes of *A*. *indicus* isolates provided publicly and generated in this study. As for the *A*. *indicus* type strain A648 three genome sequences under different strain labels (ANC 4215, CIP 110367 and DSM 25388) and accession numbers were available in the database, they were all included in the analysis. The tree was constructed using the maximum likelihood method. Bootstrap values (> 50%) after 1,000 simulations are shown at branch nodes. GenBank accession nos. are given in parenthesis. Bar, 0.05 (A) / 0.01 (B) nucleotide substitutions per site. Results of pairwise distance calculation are provided in S2 Table.
(EPS)
