# Supplementary material for: Carbapenem-resistance and pathogenicity of bovine Acinetobacter indicus-like isolates
Source: PLoS One. 2017 Feb 16;12(2):e0171986. doi: 10.1371/journal.pone.0171986 (PMC5313175; doi:10.1371/journal.pone.0171986)
Supplement: S1 Table — (DOCX) [file pone.0171986.s001.docx]

**S1 Table:** Primers and their positions used for mapping of the *bla*_OXA-23_ genetic region in bovine *A. indicus*-like isolates IHIT27599 and IHIT27630.

| **PCR** | **Primer name** | **Primer name Fig. 3** | **Primer sequence (5´**🡪**3´)** | **Tm (°C)** | **Amplicon size (bp)** | | **Position in reference sequence** | **Primer reference** |
| --- | --- | --- | --- | --- | --- | --- | --- | --- |
|  |  |  |  |  | **IHIT27599** | **IHIT27630** |  |  |
| 1 | TnpA-LEFT | 1A | TGTGTTGATATTCCTGCTTCC | 55.9 | no amplicon | 1404 | KU833219.1 (3173-3193) | This study |
|  | TnpA-RP | 1B | CAACTCTGTGCATCAGATGA | 55.5 |  |  | KU833219.1 (4576-4557) | This study |
| 2 | TnpA-FP2 | 2A | AGCTTGGTCTTTCATCAGCG | 58.6 | no amplicon | 649 | CP015364.1 (405154-405173) | This study |
|  | OXA-23-ctg69-RP | 2B | TGTCCTTGAACAATCTGACTC | 55.5 |  |  | KM977710.1 (32329-32349) | This study |
| 3 | OXA-23-FWD | 3A | AACCCCGAGTCAGATTGTTC | 57.5 | 1184 | 1184 | HQ700358.2 (10566-10547) | Groebner *et al*.[^1^](#_ENREF_1) |
|  | ATPase-univ-R | 3B | GCAACCGTCGACATCTGTTA | 58.3 |  |  | HQ700358.2 (9383-9402) | This study |
| 4 | ATPase-intern1 | 4A | TCAGGAAGATCGGACAGATC | 56.2 | 770 | 770 | HQ700358.2 (9227-9208) | This study |
|  | ISAcra1-MerR-Rev | 4B | CGCCTTTACGATGTAGGCTA | 57.2 |  |  | AJ486856.1 (9420-9439) | This study |
| 5 | IS26-FP | 5A | ACTGTTGCAAATAGTCGGTGG | 58.8 | 1359 | no amplicon | LT594095.1 (2686368-2686348) | This study |
|  | OXA-23-ctg69-RP | 2B | TGTCCTTGAACAATCTGACTC | 55.5 |  |  | KM977710.1 (32329-32349) | This study |

**Reference**

1. Groebner S, Linke D, Schutz W *et al.* Emergence of carbapenem-non-susceptible extended-spectrum beta-lactamase-producing *Klebsiella pneumoniae* isolates at the university hospital of Tubingen, Germany. *J Med Microbiol* 2009; **58**: 912-22.
