# Supplementary material for: Carbapenem-resistance and pathogenicity of bovine Acinetobacter indicus-like isolates
Source: PLoS One. 2017 Feb 16;12(2):e0171986. doi: 10.1371/journal.pone.0171986 (PMC5313175; doi:10.1371/journal.pone.0171986)
Supplement: S3 Table — CI, confidence interval. (DOCX) [file pone.0171986.s003.docx]

**S3 Table.** Median lethal doses (LD50) of *Acinetobacter* spp. injected into *G. mellonella* larvae at 24 hours post infection.

| ***Acinetobacter* strain** | **log LD_50_** | **95% CI** |
| --- | --- | --- |
| *A. baumannii* ATCC 17978 | 4.72 | 4.42 - 5.01 |
| *A. lwoffii* ATCC 15309 | 5.67 | 5.51 - 5.82 |
| *A. indicus*-like IHIT27630 | 6.20 | 5.91 - 6.50 |
| *A. indicus*-like IHIT27599 | 6.84 | 6.65 - 7.04 |

CI, confidence interval
